# Predictive Value of First-Trimester Glycosylated Hemoglobin Levels in Gestational Diabetes Mellitus: A Chinese Population Cohort Study

**DOI:** 10.1155/2021/5537110

**Published:** 2021-04-09

**Authors:** Jianbin Sun, Sanbao Chai, Xin Zhao, Ning Yuan, Jing Du, Yufang Liu, Zhi Li, Xiaomei Zhang

**Affiliations:** ^1^Department of Endocrinology and Metabolism, Peking University International Hospital, Beijing 102206, China; ^2^Department of Obstetrics and Gynecology, Peking University International Hospital, Beijing 102206, China

## Abstract

This study was aimed at exploring the predictive value of first-trimester glycosylated hemoglobin (HbA1c) levels in the diagnosis of gestational diabetes mellitus (GDM). A total of 744 pregnant women registered at the Peking University International Hospital between March 2017 and March 2019 were included in this study. Data on personal characteristics and biochemical indicators of the pregnant women were collected during the first trimester. The International Association of Diabetes and Pregnancy Study Groups has adopted specific diagnostic criteria as the gold standard for the diagnosis of GDM. Receiver operating characteristic (ROC) curve statistics were used to assess the predictive value of first-trimester HbA1c levels in the diagnosis of GDM. HbA1c levels in the first trimester were significantly higher in the GDM group than in the non-GDM group (5.23% ± 0.29% vs. 5.06 ± 0.28%, *P* < 0.05). The first-trimester HbA1c level was an independent risk factor for gestational diabetes. The area under the ROC curve (AUC) of HbA1c for GDM was 0.655 (95% confidence interval 0.620-0.689, *P* < 0.001). The positive likelihood ratio was the highest at HbA1c = 5.9%, sensitivity was 2.78, and specificity was 99.83%. There was no statistical difference in AUC between fasting blood glucose and HbA1c (*P* = 0.407). First-trimester HbA1c levels can be used to predict GDM. The risk of GDM was significantly increased in pregnant women with first‐trimester HbA1c levels > 5.9%. There was no statistical difference between first-trimester HbA1c and fasting blood glucose levels in predicting GDM.

## 1. Introduction

Gestational diabetes mellitus (GDM) is defined as abnormal glucose tolerance with onset or first recognition during pregnancy; however, blood glucose levels in cases of GDM do not reach those indicating obvious diabetes mellitus [1]. With the current global coronavirus disease 2019 (COVID-19) pandemic, local lockdowns have induced an unhealthy diet, physical inactivity, and increased psychological stress [2]. That is an even greater challenge for GDM management. Although pregnant women with GDM followed up as usual during the COVID-19 pandemic lockdown, their diabetes control was lower, with a higher rate of insulin therapy [3]. Pregnant women with GDM have an increased risk of developing preeclampsia, increased rates of cesarean sections, and an increased risk of macrosomia [4]. In addition, pregnant women with GDM have a significantly increased risk of developing type 2 diabetes mellitus later in life [5, 6]. There is a critical period for fetal organ development in the early stages of pregnancy. Abnormal glucose metabolism during this period can result in organ malformation in the developing fetus [7]. Therefore, early screening for GDM is critical. The first-trimester HbA1c level is a reliable predictor of complications during pregnancy, including preeclampsia, fetal macrosomia, and large for gestational age birth weight [8]. Fasting blood glucose (FBG) is used as an early screening tool for gestational diabetes. However, FBG requires fasting, and as FBG has great variability and poor repeatability, it is not effective in the early screening for GDM. Measuring glycosylated hemoglobin (HbA1c) levels has several advantages over measuring FBG levels [9]: it is more convenient as fasting is not required and more stable and is subject to fewer day-to-day variations due to stress or illness. HbA1c has been widely used in the diagnosis and management of diabetes patients, but its use in the diagnosis of gestational diabetes remains controversial as HbA1c levels fall during the first trimester [10]. This study was aimed at exploring the value of first-trimester HbA1c levels in predicting GDM.

## 2. Materials and Methods

### 2.1. Participants

This was a prospective cohort study. A total of 744 pregnant women registered at the Peking University International Hospital in China between March 2017 and March 2019 were included in this study. Inclusion criteria were as follows: pregnant women aged 19–45, resident in Beijing for more than 5 years and registered at this hospital, pregnancy confirmed by ultrasound or blood human chorionic gonadotropin test, and available data on first-trimester HbA1c levels. Exclusion criteria were as follows: absence of HbA1c and routine blood tests in the first trimester; absence of height and/or weight data in the first trimester; a history of prepregnancy diabetes or impaired glucose tolerance; abortion; twin or multiple births; anemia; personal or family history of thyroid disease; use of oral contraceptives or any other drug that may affect thyroid function; and presence of Hashimoto's disease, chronic autoimmune disease malignant tumors, or blood diseases.

The study was approved by the Biomedical Ethics Committee of the Peking University International Hospital (2016-015, 20160710) (2017-021, 20170608). Participants selected for the study gave their informed consent in writing before enrollment.

### 2.2. Methods

In this study, 744 pregnant women were included for follow-up during pregnancy. All participants underwent blood tests in their first trimester, including evaluation of the red blood cell (RBC) count and hemoglobin (Hb), HbA1c, FBG, triglyceride (TG), total cholesterol (TC), high-density lipoprotein cholesterol, low-density lipoprotein cholesterol, creatinine (Cr), uric acid (UA), thyroid stimulating hormone (TSH), free triiodothyronine (FT3), free thyroxine (FT4), total triiodothyronine (TT3), and total thyroxine (TT4) levels. Gestational age was confirmed on the basis of the self-reported date of the last menstrual period or by ultrasound. The nurse recorded each participant's age, number of deliveries, blood pressure, height, and weight. Participants received routine antenatal care throughout their pregnancies, and all participants were screened for gestational diabetes using a 75 g oral glucose tolerance test between 24 and 28 weeks of pregnancy.

### 2.3. Diagnostic Criteria for GDM

GDM was diagnosed using the IADPSG diagnostic criteria [11], which involves a 75 g oral glucose tolerance test (OGTT). GDM is excluded on the basis of FBG < 5.1 mmol/l, blood glucose 1 hour after glucose load < 10.0 mmol/l, and blood glucose 2 hours after glucose load < 8.5 mmol/l. GDM may be diagnosed if any blood glucose level reaches or exceeds the above limits. These diagnostic criteria were recommended by the American Diabetes Association (ADA) [12] and the Chinese Diabetes Association [13]. The diagnostic criteria for diabetes mellitus were adopted by the World Health Organization in 1999. Prepregnancy diabetes is defined as type 1 diabetes mellitus, type 2 diabetes mellitus, or a special type of diabetes diagnosed before pregnancy.

HbA1c was detected using a G8 automatic HbA1c analyzer with high-performance liquid chromatography. Thyroid function was determined using the Roche COBASE601 automatic electrochemical luminescence method.

### 2.4. Statistical Analysis

Data analysis was performed using SPSS 23.0. The Kolmogorov–Smirnov test (K–S test) was used to test the normality of distribution, and measurement data were represented as $\bar{\text{ x}}\pm s$. An independent sample *t*-test was used for comparison between the two groups according to normal distribution. The rank sum test was used to compare the two groups that did not conform to a normal distribution. Categorical variables were analyzed using the *χ*^2^ test. Logistic regression analysis was used to analyze the risk factors for GDM. MedCalc statistical software was used to analyze the receiver operating characteristic (ROC) curve of HbA1c in diagnosing GDM. The areas under the three ROC curves of FBG, HbA1c, the combination of FBG and HbA1c were compared. Statistical significance was set at *P* < 0.05.

## 3. Results

### 3.1. Comparison of General Clinical Data between the GDM Group and the Non-GDM Group

All 744 participants underwent a 75 g OGTT during the second trimester. Among them, 144 participants were diagnosed as having GDM, and 600 participants had normal blood glucose levels. The prevalence of GDM was 19.7%. The average age of the participants diagnosed with GDM was higher than that of the participants with normal blood glucose levels (32.76 years ± 3.91 years vs. 30.62 years ± 3.64 years, *P* < 0.05). First-trimester HbA1c levels were significantly higher in the GDM group than in the non-GDM group (5.23% ± 0.29% vs. 5.06% ± 0.28%, *P* < 0.05). The FBG level in the GDM group was higher than that in the non-GDM group (5.05 mmol/l ± 0.44 mmol/l vs. 4.88 mmol/l ± 0.34 mmol/l, *P* < 0.05). The incidence of gestational diabetes in multiparous participants was 26.4%, which was significantly higher than that in the primiparous participants (*P* < 0.05). Triglyceride, cholesterol, and UA levels in the GDM group were also significantly higher than those in the non-GDM group (*P* < 0.05). There were no statistically significant differences in TSH, TT3, TT4, FT3, FT4, or Cr between the two groups (Table 1).

### 3.2. Independent Risk Factors for GDM

As shown in Table 2, logistic regression analysis was performed with GDM as the dependent variable and age; BMI ≥ 24 kg/m^2^ (dichotomous variable); parity; and first-trimester HbA1c, TC, TG, LDLC, HDLC, TSH, TT3, TT4, FT3, and FT4 levels as independent variables. The results showed that age, BMI ≥ 24 kg/m^2^, first-trimester HbA1c level, and FBG level were independent risk factors for GDM.

### 3.3. ROC Curve of First-Trimester HbA1c Level for Predicting GDM

**Figure 1:**
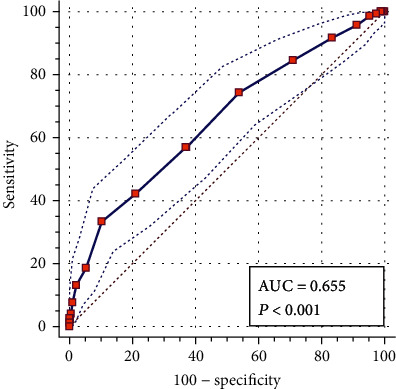
The ROC curve of first-trimester HbA1c level in the diagnosis of GDM in pregnancy.

As shown in Figure 1, the AUC of the first-trimester HbA1c level in the diagnosis of GDM was 0.655 (95% confidence interval, 0.620–0.689), *P* < 0.001. When the HbA1c level was 5.3%, the Jorden index was the highest, and the sensitivity and specificity of the diagnosis of GDM were 33.33% and 89.67%, respectively. When the HbA1c level was 5.9%, the positive likelihood ratio was the highest at 16.35, and the sensitivity and specificity for diagnosing GDM were 2.78% and 99.83%, respectively. When HbA1c was 4.4%, the negative likelihood was the lowest, sensitivity was 100%, and specificity was 1.67%, as shown in Table 3.

### 3.4. Comparisons between the AUCs of HbA1c and FBG

**Figure 2:**
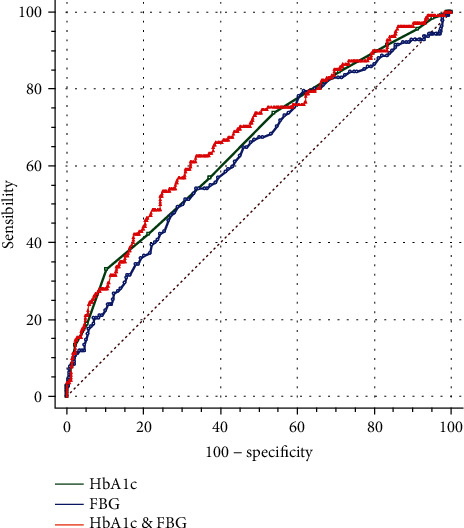
Comparison of the ROC curves of HbA1c, FBG, and the two combined indexes.

The AUC of the FBG level for the diagnosis of GDM was 0.625 (95% confidence interval, 0.589–0.660, *P* < 0.001). There was no statistical difference in the AUCs between FBG and HbA1c levels (*P* = 0.407). The AUC of the combination of FBG and HbA1c levels for the diagnosis GDM was 0.677 (95% confidence interval, 0.642–0.71, *P* < 0.001). The AUC was not significantly different between the single HbA1c level and the FBG and HbA1c (*P* = 0.145) levels combined, as shown in Figure 2.

## 4. Discussion

GDM may increase adverse outcomes such as hypoglycemia, fetal death, neonatal respiratory distress syndrome, and giant shoulder dystocia [14, 15]. The rate of cesarean section increases in pregnant women with GDM. The rate of cesarean section does not decrease even when labor is actively induced at 38 weeks' gestation [16]. GDM may increase the risk of type 2 diabetes in both the mother and her child [17]. The results of the Hyperglycemia and Adverse Pregnancy Outcome study showed that maternal blood glucose levels were continuously associated with increased birth weight, increased cord blood serum C-peptide levels, and perinatal complications, without corresponding blood glucose turning points [14]. Lifestyle interventions before 20 weeks' gestation in pregnant women at high risk of GDM can reduce the complications of GDM [18]. Therefore, early identification and active management of labor are particularly important in reducing the adverse outcomes of GDM.

Early predictors of GDM include blood glucose indicators, inflammatory markers, insulin resistance indicators, and adipocyte factors [19]; however, the latter has not been widely used in clinical practice. Blood glucose and glycosylated hemoglobin are the most commonly used indicators in clinical practice. The American College of Obstetricians and Gynecologists (ACOG) recommends a two-step GDM screening beginning with the 50 g oral glucose challenge test (OGCT), whereas the ADA recommends 75 g OGTT one-step or two-step screening for GDM [20]. A 50 g OGCT can also be used to predict delivery weight for gestational age [21, 22]. HbA1c reflects the three-month average blood glucose level, which has low individual variability and cannot be affected by time, diet, emotion, and stress responses. However, HbA1c is not recommended for the diagnosis of GDM. This study was aimed at exploring the predictive value of first-trimester HbA1c levels in patients with GDM. Although some studies have discussed the relationship between HbA1c and GDM in early pregnancy, most of these have used the two-step method as the diagnostic standard for GDM [23, 24]. In this study, a one-step method was used as the gold standard for diagnosing GDM. This study found that first-trimester HbA1c levels can be used to predict the occurrence of GDM.

This study showed that the prevalence of GDM was 19.4% and identified maternal age and BMI as risk factors for GDM. With each 1-year increase in age, the risk of GDM increased by 7.5%. Furthermore, the results of this study showed that the incidence of GDM in primiparous women was significantly lower than that in multiparous women, with a statistically significant difference between the two groups. However, after adjusting for age, it was found that parity was not a risk factor for the occurrence of GDM, which might be related to the older age of multiparous women (33.09 years ± 3.45 years vs. 29.67 years ± 3.19 years, *P* < 0.001).

It was also found that the first-trimester HbA1c level was an independent risk factor for GDM. The higher the first-trimester HbA1c level, the greater the risk of GDM. This finding is consistent with previous research [23, 24]. The reliability of using first-trimester HbA1c levels to diagnose GDM was statistically significant, with a cutoff point of 5.3% (*P* < 0.001). O'Shea et al. [25] studied trimester-specific reference intervals for HbA1c in nondiabetic Caucasian pregnant women and found the normal pregnancy HbA1c-specific reference level to be 4.3%–5.4%, which approaches the 5.3% HbA1c cutoff point. However, if 5.3% HbA1c was used as the diagnostic cutoff point for GDM, the sensitivity was 33.33%. This low sensitivity results in a high rate of missed diagnoses of GDM. Some studies have also shown that the diagnosis of GDM using HbA1c levels in early pregnancy cannot be characterized by both high sensitivity and high specificity [26, 27].

Although first-trimester HbA1c levels cannot be used to diagnose GDM directly, the high specificity of the ROC curve is helpful in predicting the occurrence of GDM. On the basis of this study, the positive likelihood ratio was highest at 5.9% HbA1c in the first trimester, and the sensitivity and specificity of GDM diagnosis were 2.78% and 99.83%, respectively, as shown in Table 3. This indicates that the rate of false diagnosis of GDM was very low in pregnant women with HbA1c > 5.9%. For these women, GDM can be diagnosed in the first trimester without waiting for an OGTT in the second trimester. In Indian pregnant women, HbA1c ≥ 5.9% as the diagnostic cutoff point also showed a low sensitivity of 1.19% and a high specificity of 99.76%[26]. HbA1c ≥ 5.9% in early pregnancy is associated with an increased risk of adverse pregnancy outcomes [28, 29]. This indicates that lifestyle interventions for pregnant women with first‐trimester HbA1c levels > 5.9% must be implemented as early as possible to reduce the likelihood of adverse pregnancy outcomes.

In this study, the negative likelihood ratio was the lowest at 4.4%, with sensitivity and specificity of 100% and 1.67%, respectively. This indicates that the risk of GDM at HbA1c < 4.4% was extremely low, and GDM can be excluded in pregnant women with first‐trimester HbA1c < 4.4%. Nevertheless, this study showed that OGTT is still recommended for screening for gestational diabetes in pregnant women with HbA1c between 4.4% and 5.9%.

The results of this study showed that the AUC of HbA1c levels did not better predict GDM than did FBG levels and that the combination of FBG and HbA1c levels did not improve the AUC. This may be related to the pathophysiological mechanisms of GDM. The occurrence of GDM is affected by various factors. In contrast to the pathogenesis of diabetes, gestational diabetes is closely related to endocrine function, substance metabolism, and the transport function of the placenta [30]. In this study, HbA1c reflects blood glucose levels before pregnancy and during first-trimester pregnancy, when placental function is still immature. For this reason, the sensitivity of HbA1c in predicting GDM is poor.

This study is a self-sequenced longitudinal prospective study focusing on first-trimester HbA1c levels in China. This study has some limitations. First, HbA1c is associated with ethnicity, and therefore, this study is only representative of eastern Asian pregnant women. Second, the influence of genetic factors and a family history of diabetes on the development of GDM in the research participants is not reflected in this study, as records of family history of diabetes were not available.

## 5. Conclusions

In conclusion, first-trimester HbA1c levels show low sensitivity and high specificity in the diagnosis of GDM and thus have limited value in diagnosing GDM. However, HbA1c levels show good predictive value for GDM. GDM can be excluded in pregnant women with first‐trimester HbA1c levels < 4.4%. However, the risk of GDM increases significantly in pregnant women with first‐trimester HbA1c levels > 5.9%. Pregnant women with a first‐trimester HbA1c level > 5.9% should be referred for lifestyle interventions in the first trimester to reduce the risk of developing GDM later in pregnancy.

## Figures and Tables

**Table 1:** Comparison of general clinical data between the GDM group and the NGDM group ($\bar{x}\pm s$).

	GDM	NGDM	*P*
*n*	144 (19.4%)	600 (80.6%)	
Age (year)	32.76 ± 3.91	30.62 ± 3.64	<0.05
Gestational week (weeks)	8.49 ± 2.15	8.66 ± 2.10	0.394
Parity			
Primiparity	72 (15.3%)	399 (84.7%)	<0.05
Multiparity	72 (26.4%)	201 (73.6%)	
SBP (mmHg)	109.63 ± 12.50	109.50 ± 11.43	0.732
DBP (mmHg)	66.14 ± 11.80	65.55 ± 10.83	0.648
BMI (kg/m^2^)	22.32 ± 2.87	21.63 ± 2.77	<0.05
BMI < 24 kg/m^2^, n/N%	92 (15.6%)	497 (84.4%)	<0.05
BMI ≥ 24 kg/m^2^, n/N%	52 (33.5%)	103 (66.5%)	
HB (g/l)	130.92 ± 8.53	130.74 ± 10.76	0.785
HbA1c (%)	5.23 ± 0.29	5.06 ± 0.28	<0.05
FBG (mmol/l)	5.05 ± 0.44	4.88 ± 0.34	<0.05
Cr (mmol/l)	48.34 ± 7.60	48.78 ± 6.96	0.871
UA (mmol/l)	266.07 ± 50.71	213.07 ± 49.38	<0.05
TC (mmol/l)	4.09 ± 0.90	3.93 ± 0.65	<0.05
TG (mmol/l)	1.11 ± 0.77	0.98 ± 0.582	<0.05
LDLC (mmol/l)	2.12 ± 0.55	2.04 ± 0.53	0.155
HDLC (mmol/l)	1.50 ± 0.81	1.42 ± 0.25	0.868
TSH (*μ*IU/ml)	1.91 ± 1.25	2.05 ± 6.74	0.228
FT4 (pmol/l)	16.79 ± 2.27	17.13 ± 2.96	0.317
FT3 (pmol/l)	4.71 ± 0.46	4.77 ± 1.61	0.822
TT4 (nmol/l)	122.70 ± 23.91	121.75 ± 23.88	0.945
TT3 (nmol/l)	2.08 ± 0.40	2.05 ± 0.45	0.393
OGTT			
FBG (mmol/l)	4.89 ± 0.55	4.48 ± 0.36	<0.05
1hBG (mmol/l)	9.67 ± 1.56	7.30 ± 1.34	<0.05
2hBG (mmol/l)	8.40 ± 1.66	6.64 ± 0.98	<0.05

Abbreviations: BMI: body mass index; SBP: systolic blood pressure; DBP: diastolic blood pressure; FBG: fasting blood glucose; HbA1c: glycosylated hemoglobin; Cr: creatinine; UA: uric acid; TC: total cholesterol; TG: triglyceride; LDL-C: low-density lipoprotein cholesterol; HDL-C: high-density lipoprotein cholesterol; TSH: thyroid stimulating hormone; FT3: free triiodothyronine; FT4: free thyroxine; TT3: total triiodothyronine; TT4: total thyroxine.

**Table 2:** Logistic regression analysis of influencing factors of GDM.

	*B*	SE	*P*	OR	95% CI
HbA1c	1.756	0.408	<0.05	5.787	2.601-12.879
Age	0.072	0.030	<0.05	1.075	1.014-1.140
Parity	0.311	0.221	0.159	1.364	0.885-2.103
BMI ≥ 24 kg/m^2^	0.115	0.035	<0.05	1.122	1.047-1.202
FBG	0.888	0.283	<0.05	2.431	1.395-4.236
UA	0.003	0.002	0.180	1.003	0.999-1.007
TC	0.144	0.149	0.334	1.155	0.862-1.546
TG	-0.097	0.167	0.559	0.907	0.654-1.258
Constant	-21.19	2.52	<0.05		

**Table 3:** ROC curve values of first-trimester Hba1c in the diagnosis of GDM.

HbA1c	Sensibility (95% CI)	Specificity (95% CI)	PLR (95% CI)	NLR (95% CI)
>4.4	100 (97.5-100.0)	1.17 (0.5-2.4)	1.01 (1.0-1.0)	0
>4.5	99.31 (96.2-100.0)	2.67 (1.5-4.3)	1.02 (1.0-1.0)	0.26 (0.03-1.9)
>4.6	98.61 (95.1-99.8)	4.83 (3.3-6.9)	1.04 (1.0-1.1)	0.29 (0.07-1.2)
>4.7	95.83 (91.2-98.5)	8.83 (6.7-11.4)	1.05 (1.0-1.1)	0.47 (0.2-1.1)
>4.8	91.67 (85.9-95.6)	16.67 (13.8-19.9)	1.1 (1.0-1.2)	0.5 (0.3-0.9)
>4.9	84.72 (77.8-90.2)	29 (25.4-32.8)	1.19 (1.1-1.3)	0.53 (0.4-0.8)
>5	74.31 (66.4-81.2)	46.17 (42.1-50.2)	1.38 (1.2-1.6)	0.56 (0.4-0.7)
>5.1	56.94 (48.4-65.2)	62.83 (58.8-66.7)	1.53 (1.3-1.8)	0.69 (0.6-0.8)
>5.12	56.94 (48.4-65.2)	63 (59.0-66.9)	1.54 (1.3-1.8)	0.68 (0.6-0.8)
>5.2	42.36 (34.2-50.9)	78.83 (75.3-82.0)	2 (1.6-2.6)	0.73 (0.6-0.8)
>5.3	33.33 (25.7-41.7)	89.67 (86.9-92.0)	3.23 (2.3-4.5)	0.74 (0.7-0.8)
>5.4	18.75 (12.7-26.1)	94.67 (92.6-96.3)	3.52 (2.2-5.7)	0.86 (0.8-0.9)
>5.5	13.19 (8.1-19.8)	97.67 (96.1-98.7)	5.65 (2.9-11.0)	0.89 (0.8-0.9)
>5.6	7.64 (3.9-13.3)	98.83 (97.6-99.5)	6.55 (2.6-16.6)	0.93 (0.9-1.0)
>5.7	4.17 (1.5-8.8)	99.5 (98.5-99.9)	8.33 (2.1-32.9)	0.96 (0.9-1.0)
>5.8	2.78 (0.8-7.0)	99.67 (98.8-100.0)	8.33 (1.5-45.1)	0.98 (0.9-1.0)
>5.9	2.78 (0.8-7.0)	99.83 (99.1-100.0)	16.67 (1.9-148.0)	0.97 (0.9-1.0)
>6	1.39 (0.2-4.9)	99.83 (99.1-100.0)	8.33 (0.8-91.3)	0.99 (1.0-1.0)
>6.1	0 (0.0-2.5)	100 (99.4-100.0)		1 (1.0-1.0)

Note: NLR: likelihood ratio; PLR: positive likelihood ratio.

## Data Availability

The data used to support the findings of this study are available from the corresponding author upon request.
